# Advanced Ceramics with Dual Functions of Healing and Decomposition

**DOI:** 10.3390/ma17030647

**Published:** 2024-01-29

**Authors:** Nobuhide Sekine, Yasushi Nakajima, Takahiro Kamo, Masahiro Ito, Wataru Nakao

**Affiliations:** 1 Graduate School of Engineering, Yokohama National University, Tokiwadai 79-5, Hodogaya-ku, Yokohama 240-8501, Kanagawa, Japan; 2 DAIICHI KIGENSO KAGAKU KOGYO CO., LTD., Kitahama 4-4-9, Chuo-ku, Osaka-shi 541-0041, Osaka, Japan; 3 Faculty of Engineering, Yokohama National University, Tokiwadai 79-5, Hodogaya-ku, Yokohama 240-8501, Kanagawa, Japan

**Keywords:** refurbish, remanufacturing, recycle, smart material, engineering ceramics, pest oxidation

## Abstract

This study developed advanced ceramic materials with both healing and decomposition functions using a metastable product generated under superheated steam. The developed composite material comprises ZrC particles dispersed in a yttria-stabilized zirconia (YSZ) matrix. After introducing a surface crack of approximately 120 μm on the composite specimen, it showed a complete strength recovery rate after one hour of heat treatment under superheated steam at 400 °C, while it exhibited a decomposition behavior after one hour of heat treatment in air at 400 °C. The XRD analysis of the heat-treated specimens showed that the final product was monoclinic ZrO_2_ under both steam and air conditions. In other words, full strength recovery in superheated steam was achieved by a chain reaction involving metastable intermediate products derived from H_2_O, unlike the reaction in air.

## 1. Introduction

With the spread and development of the circular economy, there is a growing demand for the construction of sustainable material systems that enable advanced resource recycling in ceramic materials [1,2,3,4,5]. According to OECD reports [6], the world population is estimated to reach 8.5 billion by 2030 and over 10 billion by 2060, and the use of primary resources is estimated to almost double from 89 Gt in 2017 to 167 Gt in 2060. In particular, non-metallic minerals are expected to be used in larger amounts than other resources (such as biomass, fossil fuels, and metals), and this trend is not expected to change.

Products manufactured in the future will have to be durable, repairable, upgradable, designed for disassembly, informative, and easy to reuse and recycle. These requirements require the development of materials that can be refurbished, remanufactured, or recycled after their primary use [7,8,9]. However, refurbishing, remanufacturing, and recycling technologies for ceramic materials are still in the research and development stages [10].

During refurbishing and remanufacturing, the manufacturer repairs or replaces defective components to restore them to a state similar to that of the new product. However, some reused components may contain defects, making it difficult to ensure their reliability. Therefore, if healing technology can be utilized, the refurbishment and remanufacturing of ceramics can be significantly expanded.

Simultaneously, advanced selective decomposition technologies are required. Ceramics are being classified as multi-materials and, therefore, advanced selective decomposition technologies, such as separating fibers from fiber composites, are required.

### 1.1. Self-Healing

Self-healing is a process by which a material detects damage and repairs it. Self-healing technology has been actively researched and developed for a wide variety of material systems [11,12,13,14,15,16,17,18,19,20,21,22], especially ceramics, utilizing oxidation reactions as self-healing functions [23,24,25,26,27,28,29,30,31,32,33].

Self-healing technology can repair the initial damage caused by machining and repair damage to used parts [34,35] and is expected to be applied to refurbishing and remanufacturing. Shi et al. proposed a technique for electrochemically repairing cracks in an Al_2_O_3_ composite comprising Ti particles by applying a constant voltage at room temperature [36]. This technology has the potential to realize advanced reuse such as in the refurbishing and remanufacturing of ceramics.

In self-healing ceramics, once self-healing is complete and an oxide is formed on the surface of the self-healing agent, the supply rate of oxygen is extremely slow, thus inhibiting excessive reactions. Once the crack is completely healed and the strength of the area is increased, a second impact causes a crack to form elsewhere. Since this area has a fresh self-healing function, self-healing occurs in the same manner as the first impact. Thus, the entire material can experience repeatable self-healing [37].

Osada et al. proposed that self-healing ceramics undergo the following elementary reactions for full-strength recovery [38]: (1) The inflammation stage, wherein the reaction is triggered by contact with external substances. (2) The repair stage, wherein fluid materials fill the cracks. (3) The remodeling stage, wherein the flowable material crystallizes and solidifies, resulting in strength development. From the perspective of fracture mechanics, the formation of a flowable material in the repair stage (2) is particularly important for complete strength recovery.

### 1.2. Decomposition

Several studies have reported selective degradation techniques for organic materials [39]; however, no such techniques have been reported for other material systems. This is because organic materials have various bonding modes based on their main chains and functional groups, whereas inorganic materials are composed of only homogeneous bonds such as ionic and covalent bonds. This homogeneous bonding prevents the selective decomposition of ceramics, which is a bottleneck for recycling.

Pest oxidation is an example of the degradation of homogeneous bonds in inorganic materials; however, it has only been reported as a drawback. Therefore, there are few reports on technologies that actively utilize these materials, such as MoSi_2_, NbAl, and TiB_2_ [40,41].

Although MoSi_2_ decomposes via pest oxidation in air, it has also been reported that oxidation under water vapor at (670 to 773) K is more suppressed than in air and does not cause pest oxidation. The formation of a volatile substance called MO_2_(OH)_2_ in this temperature range may influence this oxidation behavior [42].

The reason why we focused on ZrC as a new self-healing agent that can switch between degradation and healing functions is because ZrC undergoes characteristic oxidation in water vapor and air, as shown in the following Section 1.3 and Section 1.4, respectively.

### 1.3. Steam Oxidation of ZrC

The steam oxidation of ZrC is expected to produce reactions via OH groups, similar to those of Ti, a homologous element. Shimada et al. reported [43] the three-step oxidation of TiC under Ar/O_2_/H_2_O = 90/5/5 kPa and showed that the rate constants for the first and second steps at 300 °C were proportional to the first and second powers of the partial pressure of water vapor, respectively. The dependence of k on the water vapor pressure suggests that water may diffuse as H_2_O and OH in the first and second stages, respectively. In addition, as previously mentioned, the formation of thermodynamically unstable hydroxides, which are transition elements of the same period, has been reported during the steam oxidation of Mo carbides.

The dehydration reaction of Zr(OH)_4_ has been reported in many reports in the field of catalysis, and the dehydration reaction of Zr(OH)_4_ follows the reaction pathway given below [44].

Zr(OH)_4_·xH_2_O → Zr(OH)_4_ → t-ZrO_2_ → m-ZrO_2_ (1)

First, the dehydration of adsorbed water occurs at 100 °C, followed by the formation of tetragonal ZrO_2_ at 350 °C. Subsequently, tetragonal ZrO_2_ is formed. The tetragonal ZrO_2_ then undergoes a phase transformation to monoclinic ZrO_2_ at 470 °C.

We therefore assume that Zr(OH)_4_ will be formed in superheated steam, followed by the second stage of dehydration, as shown in chemical reaction (1), and that ZrC (zirconium carbide) will exhibit self-healing properties at temperatures above 350 °C.

### 1.4. Air Oxidation of ZrC

There have been many reports on the oxidation reaction of ZrC, but there are few reports on the oxidation behavior at relatively low temperatures as low as 400 °C, except for one by Shimada et al. [45]. Based on the oxidation mechanism of ZrC at high temperatures [46,47,48], the reaction proceeds as follows. First, O solidly dissolves in ZrC to form an oxycarbide layer on the surface. When the oxycarbide layer reaches a certain thickness, cubic or tetragonal ZrO_2_ is nucleated. The oxide film grows and undergoes a phase transformation to m-ZrO_2_ at a certain thickness. When ZrC oxidizes and becomes m-ZrO_2_, a volume expansion of approximately 1.4 times occurs.

In this study, we fabricated a prototype advanced ceramic with dual functions of healing and decomposition using ZrC and investigated its properties. Specifically, we evaluated the conditions required for self-healing produced by oxidation under superheated steam. The decomposition window caused by oxidation in air was also evaluated. By analyzing these behaviors in detail, the chemical reactions governing this function were determined.

## 2. Methods

### 2.1. Material

The specimen used in this study is a particle-dispersed composite material consisting of yttrium-stabilized zirconia (YSZ) and ZrC particles dispersed at a volume fraction of 30%. The raw materials are YSZ powder (HSY-3F, DAIICHI KIGENSO KAGAKU KOGYO CO., LTD, Osaka, Japan) with an average grain size of 0.48 μm and ZrC powder (FSZ010, DAIICHI KIGENSO KAGAKU KOGYO CO., LTD, Osaka, Japan) with an average grain size of 1.5 μm. The chemical compositions of raw materials are shown in Table 1. The YSZ and ZrC powders were mixed in isopropanol for 24 h via ball milling. The mixed powders were hot-pressed and sintered at 1350 °C in an Ar atmosphere for 1 h and under a surface pressure of 40 MPa to obtain plates.

### 2.2. Indentation, Heat Treatment, Bending Test, and XRD Analysis

To investigate the self-healing and decomposition behaviors of the specimens, three-point bending specimens were prepared and heat-treated in air and steam. The tensile surfaces of the 3-point bend specimens were mirror-finished, and the edges were chamfered at 45°.

To investigate the self-healing behavior in detail, a crack with a surface length of 120 μm was introduced in the center of the specimen. A crack was introduced by pressing an indenter into the specimen with a load of 3 kgf, using a Vickers testing machine (VMT-7; Matsuzawa Co., Ltd., Akita, Japan). A specimen with a crack introduced is referred to as a pre-cracked specimen.

For heat treatment in air, the specimens were placed in an electric furnace, heated up to 400 °C at 10 °C/min, held for 1 h, and then cooled in the furnace.

**Figure 1:**
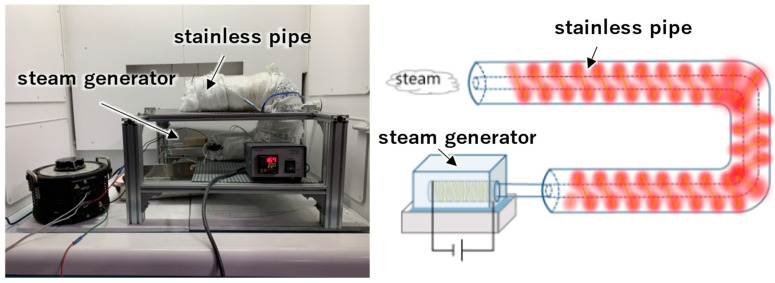
Steam generator and electric furnace.

Heat treatment in steam was performed by spraying the specimens with superheated steam at 400 °C produced by a steam generator [49]. As shown in Figure 1, the specimens were placed in a heated stainless-steel pipe and superheated steam was directed into the pipe for heat treatment. The heat treatment conditions were as follows: the specimen was exposed to steam for one hour, removed from the atmosphere, and cooled by air. 

Surface cracks were observed using a laser microscope (VK-X3000, KEYENCE CORPORATION, Osaka, Japan) before and after heat treatment to confirm the crack repair conditions. The specimens were heat-treated in air and superheated steam, as described above, and subjected to XRD analysis and SEM observations. 

A three-point bending test was performed to investigate the strength recovery behavior of the specimens after each heat treatment. The span was set to 30 mm. A universal strength-testing machine (Autograph AGS-X, SHIMADZU CORPORATION, Kyoto, Japan) was used to load the specimens at a crosshead speed of 0.5 mm/min. The load and displacement were measured simultaneously. The number of specimens was taken as N = 3 for each condition. After fracturing, the indentations on the specimens were observed using a digital microscope (VHX-2000, KEYENCE CORPORATION, Osaka, Japan) to investigate the relationship between the fracture initiation point and the pre-cracked area.

## 3. Results and Discussion

**Figure 2:**
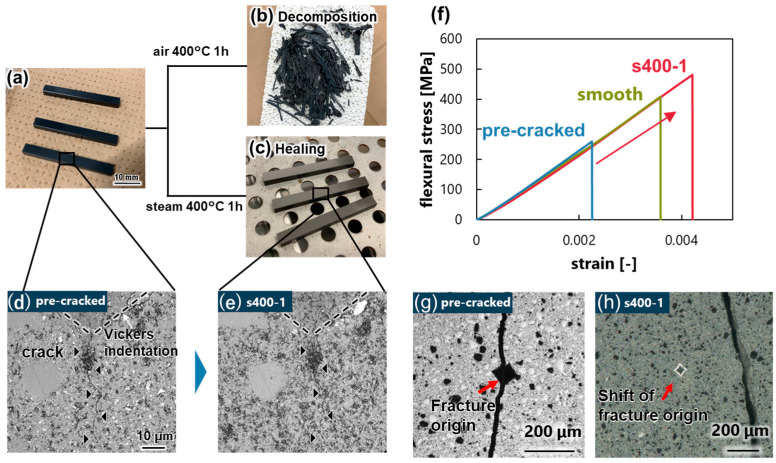
Morphology of specimen under each heat treatment condition and healing behavior. (**a**) As-received specimen, (**b**) heat-treated in air at 400 °C for 1 h, (**c**) heat-treated in superheated steam at 400 °C for 1 h, (**d**) enlarged view of crack introduced on the surface, (**e**) crack healing under superheated steam heat treatment, (**f**) strength recovery, (**g**) fracture origin of pre-cracked specimen, and (**h**) shift of fracture origin.

When the specimens were heat-treated in air and steam, there were significant differences in the final shapes, as shown in Figure 2a–c. While the specimens decomposed when heat-treated in air at 400 °C, they retained their original shapes when subjected to heat treatment in superheated steam at the same temperature of 400 °C (Figure 2c). Figure 2d,e show enlarged images of the pre-cracked area before and after heat treatment, respectively. The pre-cracks were healed via the heat treatment in steam. Figure 2f shows the healing behavior of the material. The flexural strength, which was significantly reduced by the introduction of the pre-crack, was recovered completely by heat treatment in superheated steam. The average strength of a smooth specimen was 405.61 Mpa (maximum and minimum strength was 439.09 Mpa, 369.74 Mpa, respectively), while that of a cracked specimen was 257.53 Mpa (maximum and minimum strength were 258.87 Mpa and 254.93 Mpa, respectively). The introduction of pre-cracks reduced the strength by up to 184.16 Mpa. After the heat treatment, the cracks were healed and the strength increased to an average of 486.91 Mpa (maximum and minimum strength were 529.59 Mpa and 450.26 Mpa, respectively). This was seen at the fracture initiation point, which was located at the pre-crack in the pre-cracked specimen (Figure 2g) and shifted to a location other than the pre-crack after the heat treatment (Figure 2h).

**Figure 3:**
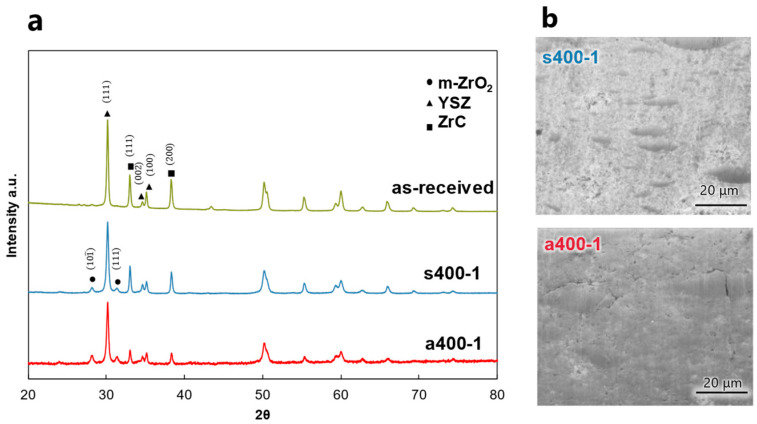
Final product under each heat treatment processes. (**a**) XRD profile; and (**b**) surface morphology.

Figure 3 shows the final products obtained in air and steam. Figure 3a shows that only monoclinic ZrO_2_ exhibited peaks after heat treatment in either air or steam. Figure 3b shows a surface SEM image of the specimen after heat treatment. Although the end products were the same, no cracks were observed on the surface after steam treatment, whereas large cracks were observed on the surface after air oxidation treatment.

The results obtained in this study satisfied the original research objective of realizing ceramics with both self-healing and decomposition functions.

**Figure 4:**
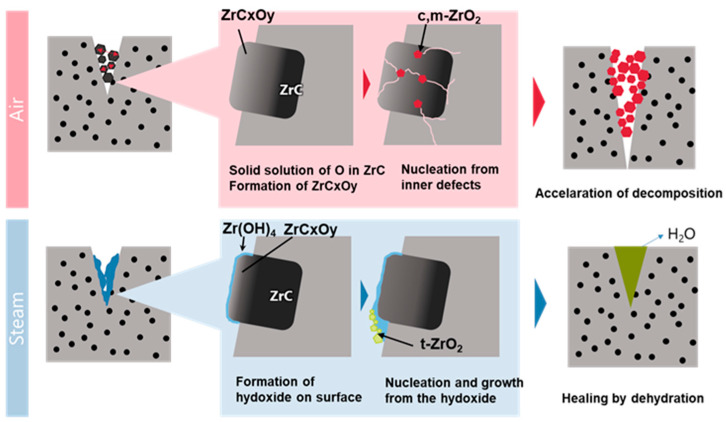
Decomposition and healing mechanism.

However, the mechanism underlying the switch between these two functions cannot be explained solely using the experimental results. Therefore, we cite the results of other studies and discuss their behavior. The proposed mechanism is illustrated in Figure 4.

Atmospheric oxidation involves a reaction similar to pest oxidation. It has been reported that in many materials that undergo pest oxidation, the main cause is the volume expansion of oxides formed at defects inside the material, such as grain boundaries [50]. Pest oxidation consists of two stages: nucleation and growth. Cracking occurs because the nucleation site is an internal defect [51].

In ZrC, oxygen solidly dissolves to form an oxycarbide. When ZrO_2_ nucleates from this oxycarbide, it does not necessarily occur on the surface; however, internal defects may become nucleation sites, as in the case of pest-type oxidation. The formation of monoclinic ZrO_2_ from ZrC was accompanied by a volume expansion of approximately 1.4 times. Therefore, when nucleation occurs in the interior, large strains accumulate and cracks form. Once cracks are formed, oxidation proceeds at an accelerated rate, and decomposition is accelerated.

Next, a first-principles study of the reactivity of ZrC with H_2_O reported that the H_2_O molecule was separated into H and OH at the surface of ZrC, forming the hydroxide Zr-OH [52]. Therefore, it is possible that the ZrC in this study also formed Zr–OH as a metastable phase during oxidation. According to Aghazadeh et al. [44], Zr(OH)_4_ slowly dehydrates with a peak at 350 °C, forming t-ZrO_2_. Then, t-ZrO_2_ undergoes a phase transformation to m-ZrO_2_ at 470 °C.

The multistep reaction via the hydroxide significantly affects the self-healing function. According to Osada et al. [38], to achieve complete strength recovery via self-healing, it is necessary to include three elementary reaction processes: (1) inflammation, (2) repair, and (3) remodeling. In particular, it is important that a flowable material is generated and that cracks are healed during the repair stage to achieve full-strength recovery.

In the reaction system used in this study, the metastable hydroxides temporarily formed by the non-equilibrium process of steam oxidation became fluid and reached the repair phase. It is also considered that this hydroxide serves as a nucleation site and thereby prevents the formation of cracks.

Therefore, by strictly controlling the water vapor partial pressure, oxygen partial pressure, and temperature, and by adjusting the rate of hydroxide formation and oxide nucleation from oxycarbide, it is possible to use self-healing and decomposition separately.

The authors believe that the results of this study are important for the realization of reuse technologies for ceramics, such as “refurbishing”, “remanufacturing”, and “advanced recycling”.

However, many technical issues remain to be resolved for the practical application of ceramic reuse technology. For example, the dynamic competition between degradation and repair is not well organized in the self-healing function, which is the subject of this study, and it is widely known that the hydrothermal degradation (LTD) of YSZ causes strength degradation [53]. Therefore, it is also necessary to analyze the kinetic competition between repair and degradation reactions, as reported in our previous studies [54].

Other issues to be solved are as follows. For practical use, it is necessary to investigate the size of cracks that can be healed and how many times they can be healed from a kinetic viewpoint. In addition, since the hydroxide could not be identified in this study, the mechanism of its formation could not be determined. We believe that the reason for the lack of identification is that the reaction occurred only near the surface, and the metastable hydroxides formed did not remain in sufficient quantities to be measured after the reaction. 

We believe that identifying the formation mechanism of metastable hydroxides and analyzing the kinetic competition will make it possible to control both the decomposition and healing functions and pave the way for the practical application of this material.

## 4. Conclusions

In this study, we developed a new ceramic material with self-healing and decomposition functions comprising ZrC dispersed in ZrO_2_. The self-healing function was activated through a heat treatment process in superheated steam at 400 °C for one hour. Initially, the average strength of a smooth specimen was 405.61 MPa, while that of a cracked specimen was 257.53 MPa. After the heat treatment, the cracks were healed and the strength increased to 486.91 MPa, indicating a complete strength recovery. The decomposition function was realized via heat treatment in air at 400 °C for one hour. The XRD results showed that the products after steam and atmospheric oxidation were identical, indicating that full-strength recovery under steam was achieved via a chain reaction involving a metastable intermediate product derived from H_2_O.

Therefore, by utilizing nonequilibrium reactions, self-healing functions can be achieved even in material systems that undergo pest oxidation, making it possible to provide a single material with both healing and decomposition functions.

## Figures and Tables

**Table 1:** Chemical composition of raw materials.

ZrC		Zr + Hf	C	Fe	Y_2_O_3_		
	wt%	87.83	12.0	<0.01	0.16		
YSZ		ZrO_2_	Y_2_O_3_	Al_2_O_3_	Fe_2_O_3_	TiO_2_	SiO_2_
	wt%	94.28	5.72	0.24	0.001	0.001	0.005

## Data Availability

The datasets generated during and/or analyzed during the current study are available from the corresponding author on reasonable request.
